# Cutaneous Effects of Notch Inhibitor Therapy: A Report of Two Cases

**DOI:** 10.1155/2020/8842242

**Published:** 2020-07-02

**Authors:** Claire J. Wiggins, Susan Y. Chon

**Affiliations:** ^1^MD Anderson Cancer Center, Department of Dermatology, 1515 Holcombe Blvd, Houston, TX 77030, USA; ^2^Baylor College of Medicine, 1 Baylor Plaza, Houston, TX 77030, USA

## Abstract

As aberrant Notch signaling has been linked to cancerous growth, Notch inhibitors represent a novel category of targeted oncological therapy. Notch pathways in tumor cells may contribute to proliferation or limit apoptosis and differentiation. Healthy skin differentiation and homeostasis are reliant on normal Notch expression, and disruption of this signaling has been implicated in dermatological conditions such as hidradenitis suppurativa, psoriasis, atopic dermatitis, and lichen planus. Here, we describe two cases of patients with cutaneous side effects from Notch inhibitor treatment for adenoid cyst carcinoma (ACC) and review the role of Notch signaling in skin disease. By illuminating connections between medication side effects and disease pathogenesis, our goal is to increase awareness of the cutaneous side effects of Notch inhibitor treatment.

## 1. Introduction

Targeted cancer therapies have brought about a paradigm shift in the field of oncology by honing in on the biochemical pathways relevant to common oncogenic mutations. Notch signaling is a highly conserved transduction pathway that is implicated in many cancers, *NOTCH* genes acting as either an oncogene or tumor suppressor depending on the cellular context [1]. Unregulated Notch signaling has been linked to hematological malignancies such as T-ALL, Hodgkin's lymphoma, and multiple myeloma, as well as solid tumors including breast, renal, lung, ovarian, prostate, and head and neck [2]. Notch may contribute to cancerous growth by preventing apoptosis and differentiation or by promoting cell proliferation [2]. Due to its role in a variety of cancers, the Notch signaling pathway represents a promising therapeutic target.

In the epidermis, Notch plays a critical role in skin development and homeostasis, with Notch activity required during the entire lifespan [1]. Mammals possess 4 different receptors, Notch 1–4, and each has demonstrated expression in the epidermis. Although the ablation of Notch 2–4 does not cause any phenotypic changes in the skin, deletion of Notch 1 receptor leads to abnormalities in the interfollicular epidermis (IFE) and hair follicle (HF), although the evidence does indicate overlap in each receptor's function [3, 4]. Notch 1 has been shown to induce differentiation of keratinocytes as well as arrest the growth of mature keratinocytes through the calcineurin-NFAT pathway [5]. The Notch pathway stimulates epidermal differentiation and promotes barrier formation [3, 6]. Retinoic acid (RA) signaling in the skin has also been directly linked to Notch 1 activity [4, 7].

When normal Notch signaling is disrupted, a number of pathologies have been documented. Inflammatory skin conditions such as hidradenitis suppurativa (HS), psoriasis, atopic dermatitis, and lichen planus have been linked to Notch dysfunction [6, 8, 9]. Notch-deficient skin has been associated with myeloproliferative disorders and *B*-lymphoproliferative disorders [6, 10]. Interestingly, a high frequency of Notch 1 receptor mutations has been identified in cutaneous squamous cell carcinoma (cSCC), indicating that Notch mutation plays an early role in skin carcinogenesis, similar to adenomatous polyposis coli mutation in colon cancer [11]. However, in head and neck SCC, over expression of Notch, rather than under expression in cases of cSCC, was linked with poorer prognosis [12, 13]. Notch 1 has been established as a tumor suppressor for both basal cell carcinoma (BCC) and cSCC [1].

Notch signaling plays a variety of roles in cutaneous tissue and contrasting roles in various tissues that warrant further study, especially as Notch inhibitor therapies for cancer are being developed. Here, we describe two cases of patients with cutaneous side effects from Notch inhibitor treatment for adenoid cyst carcinoma (ACC). We aim to draw attention to important management issues for this targeted therapy as well as gain insight into the pathogenesis of these side effects through the mechanisms of this therapy.

## 2. Case Report

### 2.1. Case #1

A 49-year-old woman receiving Notch signaling inhibitor treatment for metastatic ACC bearing activating Notch mutations presented with a rash thought to be caused by her treatment regimen with AL 101 (BMS-906024).

These erythematous hyperkeratotic papules and plaques were first noted on her antecubital fossae and bilateral lower legs. A biopsy of one of these lesions on the lower leg revealed actinic keratosis (AK), which was treated with Efudex BID for 2 weeks with good inflammatory response but without complete resolution. Over the next several weeks, she continued to develop new similar scaly papules and plaques on her legs. Patient was then treated with acitretin 10 mg daily, which led to near complete resolution of the growths.

Additionally, several months after initiation of the primary growths, she then developed erythematous subcutaneous cystic nodules on the buttock area and antecubital fossae, which were occasionally painful and pruritic. Several lesions between the buttocks drained a thick white fluid. She was prescribed doxycycline and sitz baths to treat these HS-like lesions, which helped reduce but not entirely resolve the lesions. The patient was also found to have milia on left antecubital fossa and forehead and eczematous dermatitis on the back: both likely are additional adverse effects of Notch signaling inhibitor treatment, as once the treatment was discontinued, the cutaneous symptoms abated.

### 2.2. Case #2

A 39-year-old woman presented with painful “whelps” under her breasts, axilla, and groin, which appeared in association with the initiation of treatment with a notch signaling inhibitor AL 101 (BMS-906024) for metastatic ACC bearing activating Notch mutations.

These erythematous nodules were very painful and pruritic, and several in the groin drained white fluid. She was subsequently diagnosed with HS as well as folliculitis and was prescribed oral doxycycline, topical clindamycin, and topical chlorhexidine.

Upon follow-up several weeks later, new lesions had appeared in the groin area, but the lifespan of each lesion was decreased. Patient was additionally recommended to utilize dilute bleach baths. After discontinuation of her Notch inhibitor treatment, her hidradenitis has stabilized and has been under control with a topical regimen of clindamycin and chlorhexidine.

## 3. Discussion

Our patients, both on a Notch signaling inhibitor for treatment of ACC, suffered from new onset cutaneous conditions, which were potentially caused by Notch inhibition. Neither patient had a history of skin cancer nor other significant dermatologic history. Our first patient was diagnosed with AK, HS, milia, and eczematous dermatitis. The latter patient was diagnosed with HS and folliculitis. All of these dermatologic conditions first occurred within 2 months of the initiation of Notch signaling inhibitor therapy. Of note, both patients were receiving the AL101 medication as a part of the ACCURACY clinical trial (ID: NCT03691207). NOTCH1 has been shown to control propagation and differentiation in aggressive ACC subtypes, prompting the investigation into Notch inhibiting therapies for ACC [14–16].

Due to Notch's central role in supporting the inner and outer root sheath of the HF, it is unsurprising that blockade of Notch could contribute to inflammatory diseases of the HF such as HS and folliculitis, in addition to the mechanisms of immunological dysregulation that contribute to the inflammatory reaction, fibrosis, and scarring [17]. The proposed biochemical pathogenesis of familial HS involves Notch dysfunction due to sequence variants in portions of the gamma secretase complex [17, 18]. These upstream changes result in altered HF keratinization, which causes blockage and dilation of the HF. Follicular hyperkeratosis leads to inflammation, keratin-filled epidermal cysts, abscess, and sinus tract formation [19]. In studies of Notch-deficient mice where components of the gamma secretase complex are knocked out, HFs are replaced by epidermal cysts [20]. Dysfunction of Notch has been noted in other inflammatory conditions, such as psoriasis and atopic dermatitis; however, the development of dermal cysts and follicular occlusion found in these animal knockout models lend evidence to a more specific connection between HS and Notch signaling [18].

Although data support the role of defective Notch signaling in the pathogenesis of HS, whether or not it is the driving factor of HS is still in question [17]. Importantly, knockout models with dysfunctional Notch signaling do present with HS-like lesions, but they also swiftly develop cSCC as well. This deviates from the classic HS presentation, making dysregulated Notch signaling perhaps a secondary, rather than primary, driver of HS [18]. Interestingly, in accordance with the results from animal knockout models, our patients on Notch inhibiting therapy presented here developed HS-like lesions, and one also presented with AK lesions or precursors to cSCC.

Cutaneous squamous cell carcinoma (cSCC) is the most common skin cancer with metastatic potential, representing about 20% of epithelial skin tumors. Notch 1 mutation has been established as a gatekeeper event in the development of cSCC in humans [11]. In areas of photoexposed skin, invasive cSCC lesions have been found to have decreased expression on Notch 1 [21]. Given this established connection, the development of AK in our patient on a Notch inhibiting treatment could be reasonable to expect. Although highly treatable in early stages, advanced cSCC carries a 5-year survival rate below 30%, indicating the importance of timely intervention [22, 23]. Therefore, in populations at increased risk for cSCC, proper management of these patients must include vigilant cutaneous surveillance.

Increasing clinical awareness of these potential side effects of Notch inhibitor medication can help guide safe patient management, which is important if this treatment is approved for widespread use. Currently, reports of mild gastrointestinal upset are the only documented side effects of Notch inhibitors, but our observations indicate that significant cutaneous side effects may also emerge [24]. Informing patients of potential diverse cutaneous side effects and treating them in a timely fashion will help improve overall outcomes. Learning about these side effect profiles can additionally shed light on the mechanisms of these adverse effects and open up the possibility for more rigorous studies of causation. Although the roles of Notch signaling in HS and cSCC, for example, are not fully understood, reviewing patient symptoms in the context of new therapies can continue to help describe these pathogeneses. In the case of AK and cSCC in particular, early treatment will remain central to the management of these sequelae. Here, our Notch-inhibitor patient with AK was treated successfully with acitretin, an important clinical pearl. Notch and retinoic acid signaling are interrelated, and both contribute to epidermal differentiation, so this form of side-effect management could be a logical and effective treatment for similar patients in the future [7]. This report aims to raise awareness of potential clinical effects of a treatment currently in phase II clinical trial and to encourage future study of the discussed skin lesion pathogeneses.
